# The altus health and wellness academy model: implications for philippine school health

**DOI:** 10.1016/j.pmedr.2026.103610

**Published:** 2026-08-27

**Authors:** Gilbert C. Magulod

**Affiliations:** a Professor VI, College of Teacher Education, Cagayan State University, Lasam, Cagayan Valley, Philippines; b University Director for Knowledge and Technology Management and Publication, Cagayan State University, Tuguegarao City, Philippines

Dear Editor,

I congratulate Strieter et al. (Strieter et al., 2026) for demonstrating that the Altus Health and Wellness Academy (HWA) model can improve physical activity among children living in socially vulnerable communities. More broadly, the model illustrates an important principle for Philippine school health: improving children's well-being requires interventions that address health, learning, family, and social conditions together rather than treating physical activity, nutrition, and education as separate concerns.

This whole-school, whole-child perspective is particularly relevant in the Philippines. The Second Congressional Commission on Education (EDCOM II) emphasized that learning outcomes are closely connected with children's health, nutrition, and socioeconomic circumstances (Second Congressional Commission on Education (EDCOM II), 2025). This framing provides a strong basis for considering school health programs not simply as supplementary services, but as part of the conditions that enable children to learn, participate, and thrive. The HWA model, with its integration of physical activity, nutrition education, family engagement, and sustained mentorship, offers a potentially useful framework for this broader agenda.

The Philippine nutrition context further strengthens the case for integrated school health. The 2023 Expanded National Nutrition Survey reported that approximately one in four Filipino children under five years remained stunted (Department of Science and Technology–Food and Nutrition Research Institute, 2024). Although stunting is measured in early childhood, its developmental consequences can extend into the school years. This reinforces the need for school-based approaches that complement nutritional support with opportunities for physical activity, healthy behaviors, psychosocial support, and sustained engagement with families.

Existing Philippine feeding initiatives provide an important platform for such integration. The Department of Social Welfare and Development's Supplementary Feeding Program and the Department of Education's School-Based Feeding Program provide nutritional support to vulnerable learners (Department of Social Welfare and Development, 2024; Department of Education, 2025). Building on these platforms through structured physical activity, health education, family engagement, and sustained wellness support could strengthen a whole-child approach to school health. Rather than creating parallel programs, the HWA model may therefore offer a useful conceptual reference for integrating complementary wellness components within existing school systems.

Future Philippine implementation research should examine how whole-school, whole-child wellness models can be adapted to local needs and integrated with existing feeding and school health programs. Outcomes should extend beyond physical activity to include nutritional status, school attendance, psychosocial well-being, participation, and educational outcomes. Such evidence could help inform school health policies that recognize health and education as mutually reinforcing foundations of children's development and long-term human capital.

## CRediT authorship contribution statement

**Gilbert C. Magulod:** Methodology, Investigation, Formal analysis, Data curation, Conceptualization.

## Declaration of competing interest

The authors declare that they have no known competing financial interests or personal relationships that could have appeared to influence the work reported in this paper.

## Data Availability

No data was used for the research described in the article.
